# Supplementary material for: Co-Existence of Certain ESBLs, MBLs and Plasmid Mediated Quinolone Resistance Genes among MDR E. coli Isolated from Different Clinical Specimens in Egypt
Source: Antibiotics (Basel). 2021 Jul 9;10(7):835. doi: 10.3390/antibiotics10070835 (PMC8300665; doi:10.3390/antibiotics10070835)
Supplement: Supplementary file 1 [file antibiotics-10-00835-s001.zip › antibiotics-1266631-supplementary.pdf]

Table S1. The different tested antibiotics and their different biological processes targets.

Name of Antibiotic	The Targeted Biological Process
Amoxycillin/clavulanic	Cell wall
Cephalothin	Cell wall
Cefadroxil	Cell wall
Cefotaxime	Cell wall
Ampicillin /sulbactam	Cell wall
Cefepime	Cell wall
Ceftazidime	Cell wall
Cefpodoxime	Cell wall
Sulfamethoxazole/trimethoprim	Replication
Doxycycline	Translation
Ceftriaxone	Cell wall
Aztreonam	Cell wall
Nalidixic acid	Transcription
Cefoperazone	Cell wall
Streptomycin	Translation
Tobramycin	Translation
Ofloxacin	Transcription and Replication
Gentamycin	Translation
Norfloxacin	Transcription and Replication
Ciprofloxacin	Transcription and Replication
Meropenem	Cell wall
Piperacillin /tazobactam	Cell wall
Chloramphenicol	Translation
Levofloxacin	Transcription and Replication
Amikacin	Translation and
Azithromycin	Translation
Imipenem	Cell wall

Table S2. The different thresholds of inhibition zones according to CLSI (2018).

Antibiotics	Abbreviation	Disk Potency	Diameter Zone (mm)		
			R	I	S
**Amoxycillin/clavulanic**	**AMC**	20/10 µg	≤13	14-17	≥18
Cephalothin	KF	30 µg	≤17	18-20	≥21
Cefadroxil	CFR	30 µg	≤17	18-20	≥21
Cefotaxime	CTX	30 µg	≤2217	23-25	≥26
Ampicillin /sulbactam	SAM	10/10 µg	≤11	12-14	≥15
Cefepime	FEP	30 µg	≤18	-	≥25
Ceftazidime	CAZ	30 µg	≤17	18-20	≥21
Cefpodoxime	CPD	10 µg	≤21	18-20	≥17
Sulfamethoxazole/trimethoprim	SXT	1.25/23.75 µg	≤16	11-15	≥16
Doxycycline	DO	30 µg	≤10	11-13	≥14
Ceftriaxone	CRO	30 µg	≤19	20-22	≥23

Antibiotics	Abbreviation	Disk Potency	Diameter Zone (mm)		
			R	I	S
Aztreonam	ATM	30 µg	≤17	18-20	≥21
Nalidixic acid	NA	30 µg	≤13	14-18	≥19
Cefoperazone	CEP	75µg	≤15	16-20	≥21
Streptomycin	S	10 µg	≤11	12-14	≥15
Tobramycin	TOB	10 µg	≤12	13-14	≥15
Ofloxacin	OFX	5 µg	≤12	13-15	≥16
Gentamycin	CN	10 µg	≤12	13-14	≥15
Norfloxacin	NOR	10 µg	≤12	13-16	≥17
Ciprofloxacin	CIP	5 µg	≤15	16-20	≥21
Meropenem	MEM	10 µg	≤19	20-22	≥23
Piperacillin /tazobactam	TZP	100/10µg	≤17	18-20	≥21
Chloramphenicol	C	30	≤12	13-17	≥18
Levofloxacin	LEV	5 µg	≤13	14-16	≥17
Amikacin	AK	30 µg	≤14	15-16	≥17
Azithromycin	AZ	15 µg	≤12	-	≥13
Nitrofurantoin	F	300 µg	≤14	15-16	≥14
Imipenem	IMP	10 µg	≤19	20-22	≥23

Table S3. Different serotypes of the intestinal E. coli.

Sample name	O	H
6R S	O115	H20
55S	O115	H7
5S	O115	H4
3S	O151	H2
9S	O158	H2
145S	O158	H2
178S	O158	H4
183S	O55	H5
183*S	O126	H5
81S	O86a	H2
135S	O125	H2
211S	O159	H5
130S	O157	H7
3′S	O157	H7
9′S	O157	H7

Table S4. Resistance pattern of ESBLs and MBLs producers.

Antibiotic	Resistance % *	
	ESBLs Producers (n = 85)	MBLs Producers (n = 48)
Amoxycillin/Clavulanic	99	100
Cefadroxil	95	97.9

Cephalothin	95	100
Cefotaxime	91	97.9
Ampicillin /sulbactam	90	91.6
Ceftazidime	85	93.75
Cefpodoxime	85	95.8
Doxycycline	82	81
Cefepime	80	81
Sulfamethoxazole/trimethoprim	80	85.4
Aztreonam	77	83.3
Cefoperazone	76	85.5
Ceftriaxone	74	85.4
Meropenem	69	93.75
Nalidixic acid	64	75
Streptomycin	64	62.5
Tobramycin	45	56
Gentamycin	44	39.5
Ofloxacin	44	54
Piperacillin /tazobactam	41	54
Imipenem	40	52
Ciprofloxacin	39	50
Amikacin	36	39.5
Levofloxacin	32	41.6
Chloramphenicol	29	29
Azithromycin	29	29

*percents were correlated to the number of ESBLs or MBLs producers.

Table S5. Correlation coefficient of the number of detected genes in isolates with the resistance of the antibiotics.

Antibiotic	Person Correlation Coefficient with Number of Detected Genes
Amoxycillin/clavulanic	0.189
Cefadroxil	0.283**
Cephalothin	0.384**
Cefotaxime	0.445**
Ampicillin /sulbactam	0.327
Ceftazidime	0.481**
Cefpodoxime	0.621**
Doxycycline	0.116
Cefepime	0.150
Sulfamethoxazole/trimethoprim	0.210*
Aztreonam	0.211*
Cefoperazone	0.415**
Ceftriaxone	0.424**

Nalidixic acid	0.193
Streptomycin	0.104
Tobramycin	0.180
Gentamycin	0.135
Ofloxacin	0.061
Piperacillin /tazobactam	0.385**
Imipenem	0.353**
Meropenem	0.323**
Ciprofloxacin	0.276**
Norfloxacin	0.288**
Amikacin	0.306**
Levofloxacin	0.152
Chloramphenicol	0.035
Azithromycin	0.046
MAR indexs	0.557**

p values were calculated by Fisher's exact test. * *p* value is significant at 0.05 level (2-tailed), ****p* value is significant at 0.01 level (2-tailed).

Table S6. Primers used in the current study.

Gene	Sequence	Product size	Annealing	Reference
bla-NDM	F 5'GGTTTGGCGATCTGGTTTTC-'3<br>R 5'-CGGAATGGCTCATCACGATC-3'	621 bp	55	[63]
bla-CTX-M	F 5'-TCTTCCAGAATAAGGAATCCC-3'<br>R 5'-CCGTTTCCGCTATTACAAAC-3'	909 bp	58	[64]
bla-SHV	F 5'TACCATGAGCGATAACAGCG3'<br>R 5'-GATTTGCTGATTTCGCTCGG-3	450 bp	58	[65]
bla-TEM	F 5'-TCCGCTCATGAGACAATAACC3'<br>R 5'ATAATACCGCACCACATAGCAG3'	296 bp	58	[66]
bla-IMP	F 5'GGAATAGAGTGGCTTAATTCTC3'<br>R 5'-CCAAACCACTACGTTATCT-3	188 bp	56	[67]
bla-KPC	F 5'- CAGCTCATTCAAGGGCTTTC -3'<br>R 5'- AGTCATTTGCCGTGCCATAC -3'	533 bp	58	[68]
bla-Oxa-48	F 5'-TTGGTGGCATCGATTATCGG-3'<br>R 5'-GAGCACTTCTTTTGTGATGGC-3'	743 bp	58	[69]
aac(6')-lb-cr	F 5'- TGACCTTGCGATGCTCTATG-3'<br>R 5'- TTAGGCATCACTGCGTGTTC -3'	505 bp	56	[70]
